# The Long-Term Effect of a Nine Amino-Acid Antimicrobial Peptide AS-hepc3_(48-56)_ Against *Pseudomonas aeruginosa* With No Detectable Resistance

**DOI:** 10.3389/fcimb.2021.752637

**Published:** 2021-10-05

**Authors:** Depeng Zhu, Fangyi Chen, Yan-Chao Chen, Hui Peng, Ke-Jian Wang

**Affiliations:** ^1^State Key Laboratory of Marine Environmental Science, College of Ocean and Earth Sciences, Xiamen University, Xiamen, China; ^2^State-Province Joint Engineering Laboratory of Marine Bioproducts and Technology, College of Ocean and Earth Sciences, Xiamen University, Xiamen, China; ^3^Fujian Innovation Research Institute for Marine Biological Antimicrobial Peptide Industrial Technology, College of Ocean and Earth Sciences, Xiamen University, Xiamen, China

**Keywords:** antimicrobial peptide, AS-hepc3_(48-56)_, *Pseudomonas aeruginosa*, antibiotic-resistance, membrane permeability

## Abstract

The emergence of multidrug-resistant (MDR) pathogens has become a global public health crisis. Among them, MDR *Pseudomonas aeruginosa* is the main cause of nosocomial infections and deaths. Antimicrobial peptides (AMPs) are considered as competitive drug candidates to address this threat. In the study, we characterized two AMPs (AS-hepc3_(41-71)_ and AS-hepc3_(48-56)_) that had potent activity against 5 new clinical isolates of MDR *P. aeruginosa*. Both AMPs destroyed the integrity of the cell membrane, induced leakage of intracellular components, and ultimately led to cell death. A long-term comparative study on the bacterial resistance treated with AS-hepc3_(41-71)_, AS-hepc3_(48-56)_ and 12 commonly used antibiotics showed that *P. aeruginosa* quickly developed resistance to the nine antibiotics tested (including aztreonam, ceftazidime, cefepime, imipenem, meropenem, ciprofloxacin, levofloxacin, gentamicin, and piperacillin) as early as 12 days after 150 days of successive culture generations. The initial effective concentration of 9 antibiotics against *P. aeruginosa* was greatly increased to a different high level at 150 days, however, both AS-hepc3_(41-71)_ and AS-hepc3_(48-56)_ maintained their initial MIC unchangeable through 150 days, indicating that *P. aeruginosa* did not produce any significant resistance to both AMPs. Furthermore, AS-hepc3_(48-56)_ did not show any toxic effect on mammalian cells *in vitro* and mice *in vivo*. AS-hepc3_(48-56)_ had a therapeutic effect on MDR *P. aeruginosa* infection using a mouse lung infection model and could effectively increase the survival rate of mice by inhibiting bacterial proliferation and attenuating lung inflammation. Taken together, the short peptide AS-hepc3_(48-56)_ would be a promising agent for clinical treatment of MDR *P. aeruginosa* infections.

## Introduction

Antibiotic resistance is an increasing threat to global public health. In the past few decades, the emergence of multidrug-resistant (MDR) bacteria has been one of the most serious challenges in clinical therapy. It is reported in the United States (US) that approximately 2.8 million people are infected with antibiotic-resistant pathogens each year, and more than 35,000 people die (reported in 2019) (Kadri, 2020). Accordingly, in 2017, the World Health Organization (WHO) calls for research and development of new antibiotics for 12 priority pathogens that are thought the incurable resistant bacteria and almost all antibiotics in use lose their effective activity against them. Among the antibiotics list, three Gram-negative bacteria including carbapenem-resistant and third-generation cephalosporin-resistant Enterobacteriaceae, carbapenem-resistant *Acinetobacter baumannii* and carbapenem-resistant *Pseudomonas aeruginosa* are included (Tacconelli et al., 2018). *P. aeruginosa* is an opportunistic pathogen and the main cause of nosocomial infections, especially in immunocompromised patients or intensive care unit (ICU) individuals (Maraolo et al., 2017; Moradali et al., 2017; Bassetti et al., 2018). The treatment of *P. aeruginosa* infections has become a great challenge because this pathogen can resist multiple antibiotics in clinical therapy, such as β-lactam antibiotics, fluoroquinolones, and aminoglycosides, through a variety of antibiotic-resistant mechanisms (Pang et al., 2019). Low outer membrane permeability (about 12 to 100 times lower than that of *Escherichia coli*), developed efflux systems, especially the resistance-nodulation-cell division (RND) systems, and the production of β-lactamase makes *P. aeruginosa* easily resistant to many antibiotics (Botelho et al., 2019). MDR and extensively drug-resistant (XDR) *P. aeruginosa* pose serious threats to human health and lead to high mortality. Therefore, there is an urgent need to explore new antibacterial agents to overcome antibiotic resistance. Scientists pointed out that antimicrobial peptides (AMPs) are considered as potential alternatives to conventional antibiotics (Zasloff, 2002; Mookherjee et al., 2020).

AMPs have good antimicrobial effects, and due to their rapidly bactericidal mechanism, bacteria are not easy to develop resistance (Di et al., 2020). They have increasingly emerged as a promising class of antimicrobial agents. To date, a total of 3273 AMPs (updated on 10^th^ September, 2021) have been collected in the Antimicrobial Peptide Database (https://aps.unmc.edu/), which contain both natural origin (from microorganisms to humans) and chemical synthetic products. The amino acid combinations of AMPs are diverse, but most of them are rich in cationic and hydrophobic residues (Hancock and Sahl, 2006). These two features enable AMPs to attract them to the anionic surface of bacteria through electrostatic interactions and distribute them into the membrane lipid bilayer (Brogden, 2005). Hepcidin was first isolated from human blood and urine by two independent groups in 2000 and 2001, respectively (Krause et al., 2000; Park et al., 2001). Since 2002, hepcidin genes have been isolated in various fish species, including hybrid striped bass (2002) (Shike et al., 2002), Atlantic salmon (2003) (Douglas et al., 2003), zebrafish (Shike et al., 2004), etc. Our group reported multiple hepcidin genes or variants in several marine fishes, followed by studies on the antimicrobial activities against bacteria or fungi (Ren et al., 2006; Yang et al., 2007; Wang et al., 2009; Yang et al., 2011; Cai et al., 2012; Qu et al., 2013). Unlike mammals, fish have multiple hepcidin variants, which are widely expressed in various tissues. In particular, the liver is not the only organ with high expression in humans, and fish hepcidin is also highly expressed in the kidney, spleen and intestine (Yang et al., 2007; Cho et al., 2009; Martin-Antonio et al., 2009). Several hepcidin variants that existed in a fish may be a strategy for fish to survive in the complex aquatic environment. So far, our group has identified seven hepcidin isoforms in black porgy *Acanthopagrus schlegelii* (Yang et al., 2007), among which the expression pattern of AS-hepcidin 2 and AS-hepcidin 6 *in vivo* and the antimicrobial activity *in vitro* have been characterized. AS-hepcidin 2 is predominantly expressed in liver and is significantly up-regulated upon the challenge of the bacterial mixture (including *Staphylococcus aureus*, *E. coli*, *Vibrio parahaemeolyticus*, and *Micrococcus lysodeikticus*) (Yang et al., 2011). In contrast, AS-hepcidin 6 is mainly expressed in the head kidney and trunk kidney, and is remarkably induced in the spleen and gills under the challenge of the bacterial mixture (Yang et al., 2011). Although both AS-hepcidin 2 and AS-hepcidin 6 have potent activity against bacteria and fungi tested, AS-hepcidin 6 shows a broader antimicrobial spectrum than AS-hepcidin 2 (Yang et al., 2011).

AS-hepcidin 3 (AS-hepc3) is one of the seven hepcidin isoforms. It is composed of 95 deduced amino acids and has two predicted propeptide cleavage sites (RHKR and RRRR) (Yang et al., 2007). The AS-hepc3 mature peptides (formed by RHKR or RRRR) shows low homology with the other six AS-hepcidins (38.71% ~ 51.61%), and contains more positive net charges (+ 6) than the other six isoforms (from -1 to +5). Whether AS-hepc3 with high positive charge has stronger antibacterial activity than the rest of the other six hepcidins is worthy of further study. Therefore, AS-hepc3 was selected in the study and used to evaluate its antibacterial activity against MDR bacteria, such as *P. aeruginosa*. AMPs have been widely regarded as a promising candidate against MDR bacteria (Brogden, 2005). Unlike conventional antibiotics, most of which target specific intracellular components, most AMPs interact with bacterial membranes and destroy bacterial morphology (Zasloff, 2002; Kohanski et al., 2010). For example, the membrane-permeabilizing peptide SAAP-148 (which is a truncated peptide of LL-37 from the 13^th^ to 36^th^ amino acid with partial amino acid substitutions) showed strong efficacy against MDR pathogens including *Enterococcus faecium*, *S. aureus*, *Klebsiella pneumoniae*, *A. baumannii*, *P. aeruginosa*, and Enterobacter species (de Breij et al., 2018), suggesting that it is a promising candidate for the treatment of MDR pathogen infections. Synthetic short peptides based on the sequences of natural AMPs are considered to be promising treatment options against pathogen infections. The short peptide derived from LL-37 (including the core antimicrobial region) showed similar antimicrobial and endotoxin-neutralizing activities to LL-37 (Shurko et al., 2018). The fragments of proline-rich AMP Bac5, (including Bac5_(1-31)_ and Bac5_(1-25)_), like Bac5, retained strong antimicrobial activity against *E. coli* (Mardirossian et al., 2018).

In the present study, four truncated peptides AS-hepc3_(41-71)_, AS-hepc3_(52-71)_, AS-hepc3_(41-51)_, and AS-hepc3_(48-56)_ derived from AS-hepc3 were chemically synthesized, respectively and the antimicrobial activity of each was evaluated using several pathogens. Preliminary results showed that both AS-hepc3_(41-71)_ and AS-hepc3_(48-56)_ exhibited potent antibacterial activity. Then, the mechanisms of action of the two peptides were investigated. Furthermore, a long-term comparative study on the bacterial resistance treated with AS-hepc3_(41-71)_, AS-hepc3_(48-56),_ or 12 commonly used antibiotics was in parallel carried out. In addition, the efficacy of AS-hepc3_(48-56)_ against MDR *P. aeruginosa* was evaluated using a lung infection mouse model.

## Materials and Methods

### Peptides Synthesis

Four peptides AS-hepc3_(41-71)_ (SPAGRNSRRRRCRFCCGCCPDMVGCGTCCKF), AS-hepc3_(52-71)_ (CRFCCGCCPDMVGCGTCCKF), AS-hepc3_(41-51)_ (SPAGRNSRRRR), and AS-hepc3_(48-56)_ (RRRRCRFCC) derived from AS-hepc3 (GenBank: AAU00796.2) were chemically synthesized by GL Biochem (Shanghai, China) with a purity of >95%, and verified by HPLC and mass spectrometry.

### Polyclonal Antibody Preparation

The epitope was predicted using the Optimum AntigenTM design tool. The synthesis of the selected antigenic site (SPAGRNSRRRRC), the preparation of peptide-KLH conjugated immunogen, the construction of immune host strain (New Zealand Rabbit), and the affinity purification of antibody were performed by GenScript (Nanjing, China).

### Bacterial Strains and Culture

*Pseudomonas aeruginosa* strains CGMCC 1.2421 (ATCC 9027), CGMCC 1.2387 (ATCC 27853), *P. aeruginosa* wild-type strain PAO1 CGMCC 1.12483 (ATCC 15692), *Staphylococcus aureus* strains CGMCC 1.2465 (ATCC 6538), CGMCC 1.6722 (ATCC 25923), *Escherichia coli* CGMCC 1.2389 (ATCC 11775) *Staphylococcus epidermidis* CGMCC 1.4260 (ATCC 12228), *Bacillus subtilis* CGMCC 1.3358 (ATCC 6051), *Bacillus cereus* CGMCC 1.3760 (ATCC 14579), *Shigella flexneri* CGMCC 1.1868, *Pseudomonas stutzeri* CGMCC 1.1803 (ATCC 17588) and *Acinetobacter baumannii* CGMCC 1.6769 (ATCC 19606) were purchased from the China General Microbiological Culture Collection Center (CGMCC). The clinical isolates of MDR *P. aeruginosa*, including QZ19121, QZ19122, QZ19123, QZ19124, and QZ19125, MDR *Acinetobacter baumannii* (QZ18050), MDR *Klebsiella pneumoniae* (QZ18106), and MDR *Escherichia coli* (QZ18109) were newly identified from the Second Affiliated Hospital of Fujian Medical University (Quanzhou, Fujian, China).

These new *P. aeruginosa* isolates were stored in nutrient broth supplemented with 20% (v/v) glycerol at −80°C. Before experiments, the inoculum of the frozen stocks was grown on LB agar plates at 37°C overnight. For each experiment, the bacteria were cultured in LB medium at 200 rpm at 37°C to reach the exponential phase, and diluted to the required inoculum concentration according to the optical density at 600 nm.

### Determination of the Spectrum of Synthetic Peptides

The MIC value for each peptide was determined using a microdilution method as described previously (Shan et al., 2016). Briefly, a logarithmic growth-phase culture of bacteria was diluted to ~ 10^5^ CFU/mL in 10 mM sodium phosphate buffer (NaPB, pH 7.4) supplemented with 40% Mueller-Hilton broth (MHB), and then added to the equal volume of the peptide in water (with final concentrations ranging from 1 to 32 μM) in 96-well polystyrene flat-bottom plates (NEST, China). The bacteria were exposed to an equal volume of water without peptides as an untreated control group. After 18-24 hours of stationary incubation at 37°C, the lowest concentration without visible bacteria growth was considered as the minimum inhibitory concentration (MIC) value. The bactericidal activity was determined by the minimum peptide concentration that killed ≥ 99.9% of bacteria as the minimal bactericidal concentration (MBC) value. The experiments were performed in triplicate.

### Time-Killing Kinetics

For time-killing kinetic experiments, *P. aeruginosa* PAO1 cells (~10^5^ CFU/mL) were exposed to 8 μM and 16 μM AMPs based on the MIC assay. As an untreated control, the bacteria were incubated with water instead of AMPs. At each time interval after treatment (such as 5 min, 15 min, 30 min, 60 min, and 120 min), 50 μL of the diluted suspensions were spread on Mueller-Hinton agar (MHA) plates and incubated at 37°C overnight. The number of surviving bacteria was counted. Three independent experiments were conducted.

### Scanning Electron Microscope and Transmission Electron Microscope Observation

*P. aeruginosa* PAO1 cells were cultured to the exponential growth phase and harvested. The pellets were washed and diluted in PBS to a concentration of approximately 5×10^8^ CFU/mL, treated with 24 μM peptide at 37°C for 30 min, and then collected at 3,000 g for 2 min. The samples were washed in PBS, fixed with 2.5% glutaraldehyde at 4°C overnight, and then washed again with PBS three times. The pellets were resuspended in about 10 μL PBS solution, and then deposited on a glass slide covered with polylysine. Afterward, the cells were dehydrated using graded ethanol series (30%, 50%, 70%, 80%, 95%, and 100%). Then, the samples were dehydrated in a critical point dryer (EM CPD300, Leica, Germany) and gold coated. The samples were observed with a scanning electron microscope (SEM) (FEI Quanta 650 FEG, Thermo Fisher, USA).

Bacteria for transmission electron microscope (TEM) observation samples were prepared as described above for SEM. After spinning down, the bacterial pellets were embedded in 2% agar, and the agar blocks were cut into cubes, fixed with 2.5% glutaraldehyde at 4°C overnight, and then washed three times in PBS. The samples were post-fixed with 1% osmium tetroxide, then dehydrated with gradient ethanol series, stained with uranyl acetate, rinsed in acetone, and subsequently embedded in epoxy resin. Finally, thin sections were observed using a TEM (HT-7800, Hitachi, Japan).

### Immunogold Electron Microscopy Observation

*P. aeruginosa* PAO1 cells were prepared as described above for SEM, and then the pellets were fixed with 4% paraformaldehyde in PBS for 3 h, embedded in 2% agar, and trimmed into small pieces. The samples were sectioned with an ultramicrotome (UC-7 RT, Leica, Germany) after they were fixed and dehydrated. The thin sections were incubated with rabbit antibody at room temperature for 5 min, washed with water, and then incubated with 15-nm gold particle labeled secondary antibody (Electron Microscopy Sciences, USA) for 3 h at room temperature, washed with water, finally fixed with 2.5% glutaraldehyde for 15 min at room temperature, washed with water. The untreated group was considered the control group. In addition, the sample incubated without primary antibody was used to detect non-specific binding. After drying at room temperature, the sections were stained and then observed using a TEM (HT-7800, Hitachi, Japan).

### Outer Membrane Permeability Assay

Outer membrane permeability was measured by the hydrophobic fluorescent probe N-phenyl-1-naphthylamine (NPN) (Sigma-Aldrich, USA) uptake assay as described previously with some modifications (Silva et al., 2020). Briefly, log-phase cultures of *P. aeruginosa* PAO1 cells were collected by centrifugation (3,000 g, 2 min). The pellets were washed and resuspended to approximately 10^8^ CFU/mL in 5 mM HEPES buffer (pH 7.4, containing 5 mM glucose). 10 μL of NPN from 1 mM stock solution in 95% ethanol was added to each milliliter of bacterial cells to a final concentration of 10 μM, and then they were inoculated in a 96-well black flat-bottom microplate (NUNC). The background fluorescence was measured using a microplate reader (Infinite F200 PRO, TECAN, Switzerland) at 350/420 nm excitation/emission wavelengths, respectively. After that, peptide (final concentrations of 0 μM, 4 μM, 8 μM, 16 μM, and 24 μM) or polymyxin B (PMB, 1 μg/mL) was added to each well. The time-dependent effect of peptide on NPN fluorescence was measured every 2 min. The experiments were conducted in triplicate, and three independent tests were performed.

### Inner Membrane Permeability Assay

The effect of peptide penetrating the inner membrane of *P. aeruginosa* PAO1 was measured using LIVE/DEAD BacLight™ Bacterial Viability kit L7012 (Invitrogen, USA) according to the manufacturer’s instructions. Briefly, *P. aeruginosa* PAO1 cells in the exponential phase were collected and resuspended in PBS to a final concentration of ~10^7^ CFU/mL. Each sample was incubated with various concentrations (0 μM, 4 μM, 8 μM, 16 μM and 24 μM) of peptide or polymyxin B (PMB, 1 μg/mL) at 37°C for 30min. The pellets of PAO1 were washed twice with PBS and resuspended in SYTOX 9/PI dye mixture following the instruction. The samples were incubated in the dark at room temperature for 15 min, and then analyzed by flow cytometer (CytoFLEX, Backman, USA). The data analysis was performed by CytExpert 2.3 software. Three independent experiments were performed.

### ATP Release

The extracellular ATP levels of *P. aeruginosa* treated with peptide were measured as described previously with some modifications (Khara et al., 2015). Briefly, *P. aeruginosa* PAO1 cells in the exponential phase were washed and resuspended in PBS to approximately 10^8^ CFU/mL. An equal volume of peptide solution was added to the bacterial suspension to make the final concentration of 4 μM, 8 μM, 16 μM and 24 μM respectively, and then the mixtures were incubated at 37°C for 30min. The pellets of PAO1 were centrifuged at 5,000 g for 5 min, and then the supernatant was added to an equal volume of boiling TE buffer (50 mM Tris, 2 mM EDTA, pH 7.8). The mixture was boiled for another 2 min and kept on ice until examination. The cooled mixture was added to luciferin-luciferase assay mixture from the ATP Determination Kit (Invitrogen, USA), and luminescence was detected using a Glo-Max™ 20/20 luminometer (Promega, USA). The extracellular ATP concentrations were determined according to the standard curve, which was carried out following the manufacturer’s instructions. Three biological replicates were performed in each independent experiment, and two independent experiments were performed.

### Resistance Development

The resistance of *P. aeruginosa* to antibiotics was evaluated as previously described with some modifications (Ling et al., 2015). To study the development of resistance through sequential passaging, *P. aeruginosa* PAO1 cells were harvested in the exponential phase and then diluted to approximately 1×10^6^ CFU/mL in 10 mM sodium phosphate buffer (NaPB, pH 7.4) supplemented with 40% MHB. An aliquot (50 μL) of bacterial culture was added to a 96-well polypropylene flat-bottomed plate containing an equal volume of different concentrations of AS-hepc3_(41-71)_, AS-hepc3_(48-56)_, or antibiotics, including penicillin, ceftazidime, cefepime, aztreonam, imipenem, meropenem, ciprofloxacin, levofloxacin, gentamicin, tobramycin, polymyxin B and colistin. The plates were statically cultured at 37°C for 18-24 hours. The lowest concentration of antimicrobial compound without visible bacterial growth was defined as the MIC of this day. Starting from the second-highest concentration, the cultures that significantly allowed PAO1 to grow were diluted 1,000-fold in fresh medium, and then these mixtures were incubated as described above. This serial passaging was repeated daily for 150 days. In the control group, the bacteria were incubated with water. The experiments were performed in triplicate.

### Cytotoxicity and Hemolytic Activity

A microplate MTS assay was used to evaluate the cytotoxicity of AS-hepc3_(48-56)_ to mammalian cells as previously described (Yang et al., 2020). AML12 mouse liver cell line, HEK 293T human embryonic kidney cell line, and HepG2 liver hepatocellular carcinoma cell line were obtained from Stem Cell Bank, Chinese Academy of Sciences (https://www.cellbank.org.cn/). Briefly, exponentially growing AML12 mouse liver cell line (in Dulbecco’s Modified Eagle Medium/Nutrient Mixture F-12 supplemented with 10% fetal bovine serum, 10 μg/mL human insulin, 5.5 μg/mL human transferrin, 5 ng/mL sodium selenite, and 40 ng/mL dexamethasone), HEK 293T human embryonic kidney cell line (in Dulbecco’s Modified Eagle Medium supplemented with 10% fetal bovine serum) and HepG2 cell line (in Minimum Essential Medium α supplemented with 10% fetal bovine serum) were seeded into a 96-well flat-bottomed plate (Thermo Fisher, USA) and incubated at 37°C with 5% CO_2_ overnight. Then, the medium was replaced with fresh medium containing dilutions of AS-hepc3_(48-56)_ (10 μL of two serial dilutions in PBS to 90 μL medium) with the final concentration at 0 μM, 40 μM, and 80 μM. Following 24 h incubation, cell viability was assessed using CellTiter 96^®^ AQueous Non-Radioactive Cell Proliferation Assay kit (Promega, USA). After 3 h, the absorbance at 492nm was measured using a microplate reader (Infinite F200 PRO, TECAN, Switzerland). Experiments were performed with at least five biological replicates.

The hemolytic activity of AS-hepc3_(48-56)_ was determined using fresh mouse erythrocytes. The blood was centrifuged at 500 g for 3 min, and the erythrocytes were washed three times with saline until the upper phase was clear and resuspended to 4% erythrocytes. Aliquots of the red blood cell solution were seeded into a 96-well plate containing 100 μL of AS-hepc3_(48-56)_ in different concentrations (32-512 μM) and incubated at 37°C for 1 h. After incubation, the aliquots of the supernatant were collected at 4,000 rpm for 3 min and then transferred to a flat-bottom 96-well plate. The hemoglobin released after lysis of the red blood cells was monitored at 540 nm using a microplate reader (Infinite F200 PRO, TECAN, Switzerland). The negative and positive controls for hemolysis were used as saline and 1% Triton X-100, respectively. The percentage of hemolysis was calculated using the following formula:


$$\%\text{Haemolysis}=[(A_{540}\text{test sample}-A_{540}\text{negative control})/(A_{540}\text{positive control}-A_{540}\text{negative control})]\;\times 100$$

Two independent runs of the assay were performed, and three replicates were used in each run for each concentration.

### Mouse Infection Model

The efficacy of AS-hepc3_(48-56)_ was further evaluated using a mouse model of pneumonia caused by MDR *P. aeruginosa* QZ19125 as described previously with some modifications (Suresh Kumar et al., 2014; Chen et al., 2018). The animal experiments were conducted in accordance with national guidelines and approved by the Laboratory Animal Management and Ethics Committee of Xiamen University (XMULAC20210029). Wild-type C57BL/6 male mice (n= 8), aged 4 to 6 weeks and weighing 18 to 20 g, were anesthetized with 1% pentobarbital (50 mg/kg) *via* intraperitoneal injection and instilled intratracheally with ~ 1×10^8^ CFU in 50 μL of saline. One hour after exposure, AS-hepc3_(48-56)_ (10 mg/kg, 20 mg/kg) or tobramycin (20 mg/kg) was administered intratracheally in 50 μL of saline. Control mice were instilled with 50 μL of saline without peptide. Mice were monitored every 12 h to 96 h. The cumulative survival rate was determined. The left lung was homogenized in 2 mL sterile saline. The lung homogenates were cultured on LB agar after proper dilution and incubated at 37°C for at least 18 h before counting the number of colonies. The right lung was fixed with 10% formaldehyde and embedded in paraffin for hematoxylin and eosin (H&E) staining. The *in vivo* safety of AS-hepc3_(48-56)_ was performed using wild-type C57BL/6J mice (n= 5) aged 4 to 6 weeks. The mice were anesthetized with 1% pentobarbital and instilled intratracheally with 8 mg/mL peptide in 50 μL of saline. Control mice were instilled with 50 μL of saline without peptide. The survival of the mice was monitored for 4 days.

### Statistical Analysis

Data were typically expressed as means ± standard deviations (SD). Statistical comparisons were performed using the one-way analysis of variance (ANOVA) with SPSS 18.0 software. The differences were defined as significant when *P* < 0.05.

## Results

### Antimicrobial Activity of AS-hepc3 Derived Four Synthetic Peptides

**Figure 1:**
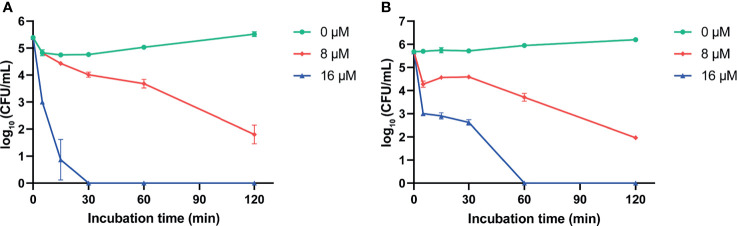
The time-killing curves of AS-hepc3_(41-71)_ and AS-hepc3_(48-56)_ on PAO1. **(A)** The time-killing curve of AS-hepc3_(41-71)_ on PAO1. **(B)** The time-killing curve of AS-hepc3_(48-56)_ on PAO1.

We evaluated the antimicrobial activity of two predicted mature peptides AS-hepc3_(41-71)_ and AS-hepc3_(52-71)_ derived from AS-hepc3 by determining their MICs and MBCs against *P. aeruginosa* PAO1. As shown in **Table 1**, AS-hepc3_(41-71)_ exhibited significant antibacterial activity against PAO1 at 8 μM and had a bactericidal effect at 16 μM. Then, we tested the antimicrobial activity of AS-hepc3_(41-71)_-derived short peptides, AS-hepc3_(41-51)_ and AS-hepc3_(48-56)_, against PAO1. We found that AS-hepc3_(48-56)_ exhibited potent antimicrobial activity against PAO1 similar to AS-hepc3_(41-71)_ (**Table 1**). In addition, AS-hepc3_(41-71)_ and AS-hepc3_(48-56)_ also showed strong antimicrobial activity against *S. aureus*, *S. epidermidis*, *B. subtilis*, *E. coli*, *A. baumannii*, *S. flexneri*, *P. stutzeri*, and other *P. aeruginosa* strains with the MIC values from 2 μM to 16 μM (**Table 2**). However, even at a high concentration of 128 μM, AS-hepc3_(41-51)_ and AS-hepc3_(52-71)_ could hardly inhibit the growth of PAO1. The time-killing curves showed that 16 μM AS-hepc3_(41-71)_ or AS-hepc3_(48-56)_ had a bactericidal effect (killing rate of 99.9%) within 30 min or 60 min, respectively (**Figure 1**).

**Table 1:** The antimicrobial activity of AS-hepc3–derived peptides against *P. aeruginosa* PAO1.

Peptide	Sequence	MIC (μM)	MBC (μM)
AS-hepc3_(41-71)_	SPAGRNSRRRRCRFCCGCCPDMVGCGTCCKF	8	16
AS-hepc3_(52-71)_	CRFCCGCCPDMVGCGTCCKF	>128	–
AS-hepc3_(41-51)_	SPAGRNSRRRR	>128	–
AS-hepc3_(48-56)_	RRRRCRFCC	8	16

**Table 2:** The MIC values of AS-hepc3_(41-71)_ and AS-hepc3_(48-56)_ against different microorganisms.

Microorganism	CGMCC.NO	AS-hepc3_(41-71)_		AS-hepc3_(48-56)_	
		MIC (μM)	MBC (μM)	MIC (μM)	MBC (μM)
B. subtilis	1.3358	8	8	8	8
B. cereus	1.3760	32	32	16	16
S. aureus	1.2465	16	16	8	8
S. aureus	1.6722	8	16	8	8
S. epidermidis	1.4260	8	8	2	4
A. baumannii	1.6769	8	16	16	16
E. coli	1.2389	16	16	16	16
P. stutzeri	1.1803	2	4	2	4
P. aeruginosa	1.2421	8	16	8	16
P. aeruginosa	1.2387	8	16	8	8
S. flexneri	1.1868	8	16	8	8

### The Morphological Changes of *P. aeruginosa* After Peptide Treatment

**Figure 2:**
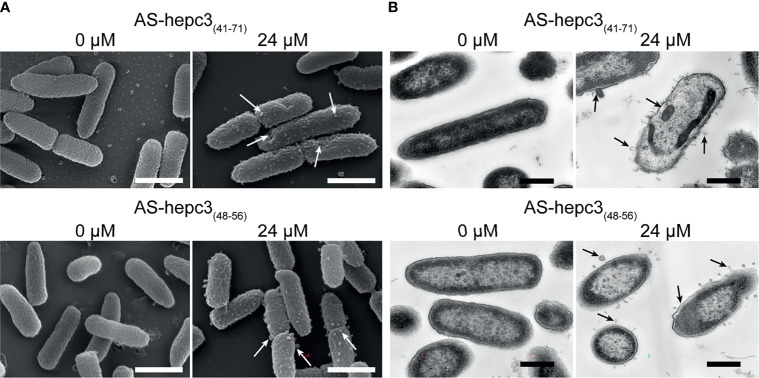
Electron micrographs of *P. aeruginosa* PAO1. **(A)** Scanning electron micrographs of PAO1, exposed to PBS, AS-hepc3_(41-71)_, or AS-hepc3_(48-56)_ at 24 μM, respectively. Scale bars, 1 μm. **(B)** Transmission electron micrographs of PAO1, exposed to PBS, AS-hepc3_(41-71)_, or AS-hepc3_(48-56)_ at 24 μM, respectively. Scale bars, 500 nm.

The morphological changes of *P. aeruginosa* after AS-hepc3_(41-71)_ or AS-hepc3_(48-56)_ treatment were observed by SEM, as shown in **Figure 2A**. Without treatment, the bacteria showed an integrity surface morphology. Compared with the control, peptides changed the permeability of bacterial membranes. After 30 min of treatment, bubbles were significantly distributed around the cell surface when treated with 24 μM AS-hepc3_(41-71)_, as well as AS-hepc3_(48-56)_. To visualize the cell envelope structure and cytoplasmic changes, we performed TEM on thin sections of *P. aeruginosa* cells after exposure to AS-hepc3_(41-71)_ or AS-hepc3_(48-56)_ for 30 min, as shown in **Figure 2B**. The bacteria cells in the control group showed an intact double-membrane cell envelope structure. However, after exposure to AS-hepc3_(41-71)_ or AS-hepc3_(48-56)_, the membrane was significantly ruptured and the intracellular contents leached out. The cytoplasm was released to the outside or around the outer membrane in the form of bubbles. Both AS-hepc3_(41-71)_ and AS-hepc3_(48-56)_ could increase the permeability of the outer membrane, destroy the inner membrane, and caused the lysis of the cytoplasm.

### The Increased Permeability of the Bacterial Outer and Inner Membrane

**Figure 3:**
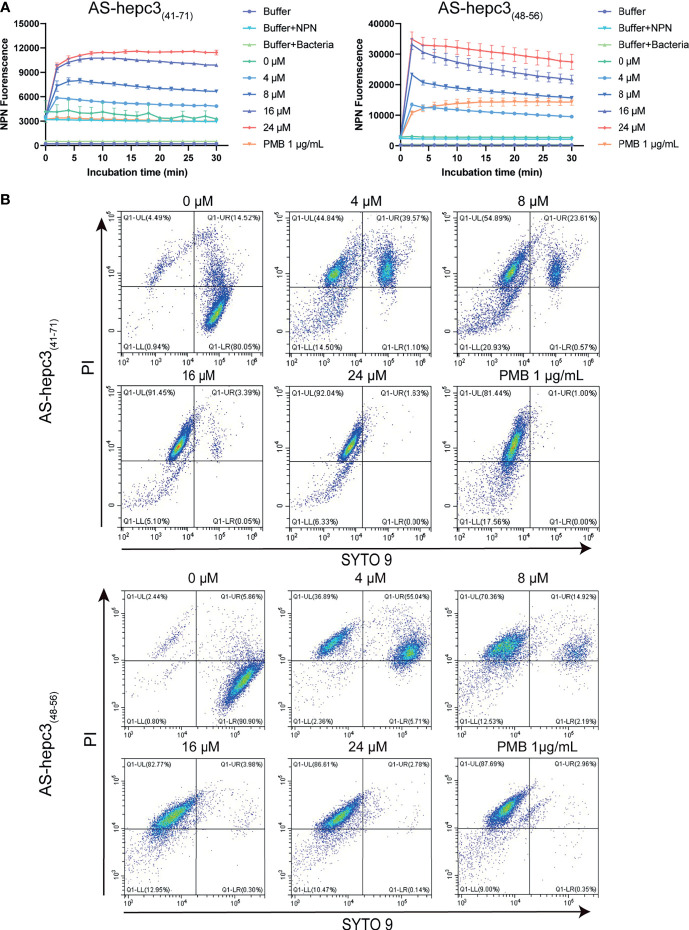
Outer and Inner membrane permeability of PAO1 after the treatment of AMPs. **(A)** Outer membrane permeability after AS-hepc3_(41-71)_ or AS-hepc3_(48-56)_ treatment. The NPN fluorescence was recorded at λex = 350 nm and λem = 420 nm. **(B)** Inner membrane permeability after AS-hepc3_(41-71)_ or AS-hepc3_(48-56)_ treatment. PMB means polymyxin B.

The outer membrane of Gram-negative bacteria plays a crucial role as a physical protective barrier. The membrane non-permeability fluorescent dye NPN (219 Da) could enter the perturbed cell membrane and interact with the phospholipid, resulting in a significant increase in fluorescence (Ma et al., 2016). As shown in **Figure 3A**, the addition of AS-hepc3_(41-71)_ or AS-hepc3_(48-56)_ caused the fluorescence intensity of NPN to increase in a dose-dependent manner due to the uptake of this hydrophobic fluorescent probe by the outer membrane of PAO1. On the other hand, the ability of these two peptides to disrupt the intact PAO1 cell inner membranes was monitored using the influx of nucleic acid stain SYTO 9 and propidium iodide (PI) to analyze the fluorescence signals by flow cytometry. The results showed that both AS-hepc3_(41-71)_ and AS-hepc3_(48-56)_ increased the inner membrane permeability in a concentration-dependent manner, as shown in **Figure 3B**. These results suggested that the integrity of the plasma membrane was damaged after peptide treatment.

### Location of AS-hepc3_(41-71)_ and AS-hepc3_(48-56)_ in *P. aeruginosa*

**Figure 4:**
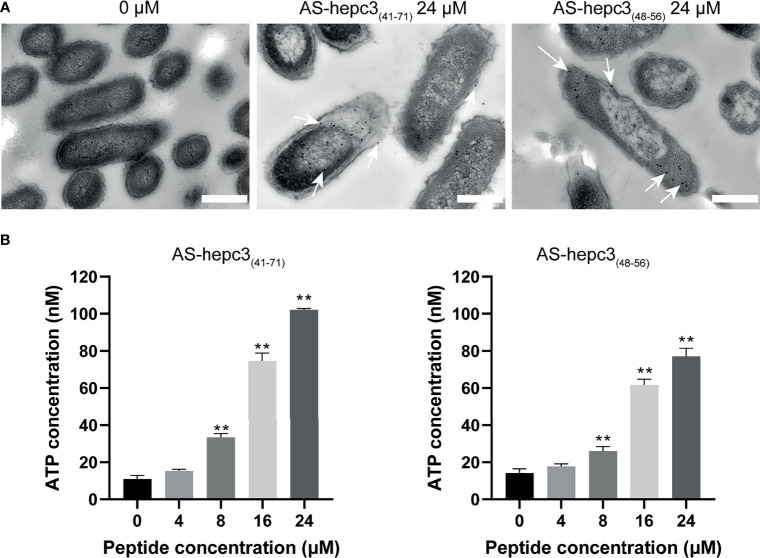
The location and effect of AS-hepc3_(41-71)_ and AS-hepc3_(48-56)_ on PAO1. **(A)** The location of AS-hepc3_(41-71)_ and AS-hepc3_(48-56)_ on PAO1. Scale bars, 500 nm. **(B)** The effect of AS-hepc3_(41-71)_ and AS-hepc3_(48-56)_ on the release of ATP at different concentrations. Error bars represent the standard deviation from the mean (n = 3), significantly different (**P < 0.01) as compared to control (0 μM), calculated using one-way ANOVA.

To accurately detect the location of AS-hepc3_(41-71)_ and AS-hepc3_(48-56)_ in bacteria, immunogold electron microscopy was used for observation. Sample images of untreated and treated bacterial cells were shown in **Figure 4A**. It was found that AS-hepc3_(41-71)_ or AS-hepc3_(48-56)_ was located at membrane and cytoplasm areas on *P. aeruginosa*. We found that a few gold signals were located near the outer membrane, and most of the gold signals were observed in the cytoplasm at a concentration of 24 μM.

### The Release of Bacterial Cytoplasmic Contents

Membrane damage leading to reduced barrier function would result in the release of ATP, a key component of cells. The extracellular ATP levels after exposure to different concentrations of AS-hepc3_(41-71)_ and AS-hepc3_(48-56)_ were evaluated. As shown in **Figure 4B**, after 30 min of incubation, the peptide-induced ATP release of PAO1 cells was concentration-dependent. The extracellular ATP level was significantly higher than that of untreated cells when the peptide concentration was greater than 8 μM.

### AS-hepc3_(41-71)_ and AS-hepc3_(48-56)_ Kill *P. aeruginosa* Without Inducing Resistance

**Figure 5:**
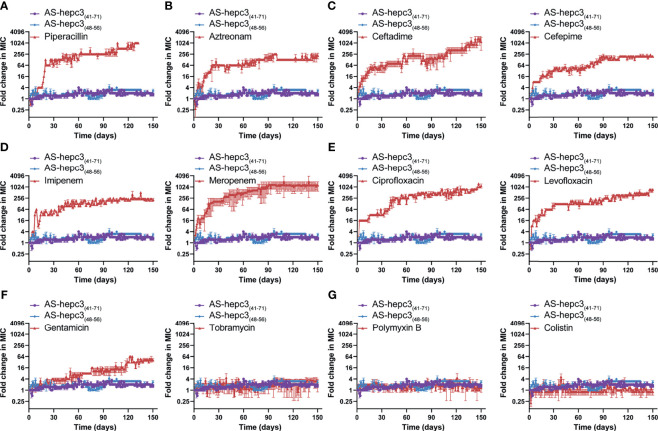
AS-hepc3_(47-71)_ and AS-hepc3_(48-56)_ kill *P. aeruginosa* PAO1 without inducing resistance selection after serial passaging treatment in the presence of sub-MIC. Antibiotics for comparisons, **(A)** Piperacillin; **(B)** Aztreonam; **(C)** Ceftazidime and Cefepime; **(D)** Imipenem and Meropenem; **(E)** Ciprofloxacin and Levofloxacin; **(F)** Gentamicin and Tobramycin; **(G)** Polymyxin B and Colistin. The y-axis indicates the fold change (in log_2_) of the minimal inhibitory concentration (MIC) relative to the MIC of the first day during passaging, and the x-axis represents the number of days. The experiment is performed in triplicate.

The emergence of drug resistance in *P. aeruginosa* is one of the main challenges in the treatment of clinical infections. In our study, the MIC values of AS-hepc3_(41-71)_ and AS-hepc3_(48-56)_ against clinical isolated MDR pathogens were from 8 μM to 32 μM, as shown in **Table 3**. In addition, we evaluated the ability of *P. aeruginosa* PAO1 to develop resistance to AS-hepc3_(41-71)_, AS-hepc3_(48-56)_, and selected 12 clinical commonly used antibiotics for comparative analysis (**Figure 5** and **Table 4**). Under the treatment of AS-hepc3_(41-71)_ or AS-hepc3_(48-56)_ at the sub-MIC level, the 150-day continuous passage of PAO1 failed to produce resistant mutants. The MIC value only increased by about 1.5-fold of AS-hepc3_(41-71)_ from the first day to the 150th day (the MIC of the first day was 8 μM, and the MIC of the last day was 12 μM). The MIC value increased by about 3-fold of AS-hepc3_(48-56)_ from the first day to the 150th day (the MIC of the first day was 8 μM, and the MIC of the last day was 24 μM). However, PAO1 developed significant resistance to 9 of the 12 selected antibiotics, including penicillin, ceftazidime, cefepime, aztreonam, imipenem, meropenem, ciprofloxacin, levofloxacin, gentamicin, and the other 3 antibiotics (tobramycin, polymyxin B, and colistin) did not produce obvious drug resistance. When cultured in the presence of meropenem for three days, a 4-fold increase in MIC was observed, and the MIC increased to 1024-fold at the 150th day (from 0.125 to 128 μg/mL). After the treatment of piperacillin, PAO1 rapidly produced resistant mutants after 19 days (MIC=128 μg/mL), and the MIC increased from 4 μg/mL to 4096 μg/mL after 128 days, an increase of 1024 times. Various other conventional antibiotics also induced resistant mutations of PAO1, including gentamicin, ceftazidime, cefepime, ciprofloxacin, levofloxacin, aztreonam. The MIC values of all antibacterial agents of the first day and the 150th day were listed in **Table 4**.

**Table 3:** Susceptibility of clinical isolated multidrug-resistant (MDR) pathogens to antibiotics, AS-hepc3_(41-71)_, and AS-hepc3_(48-56)_.

Strain	Antibiotic resistance																
	Amikacin	Ciprofloxacin	Gentamicin	Meropenem	Cefoperazone/Sulbactam	Tobramycin	Imipenem	Aztreonam	Cefepime	Levofloxacin	Piperacillin	Ceftazidime	Piperacillin/Tazobactam	AS-hepc3_(41-71)_ (μM)		AS-hepc3_(48-56)_ (μM)	
														MIC	MBC	MIC	MBC
QZ18050														16	16	16	16
QZ18106														16	32	32	32
QZ18109														8	16	8	16
QZ19121														8	16	8	16
QZ19122														8	16	8	16
QZ19123														8	8	8	8
QZ19124														8	16	8	16
QZ19125														8	32	8	16

Bacteria susceptible, intermediate, or resistant to each antibiotic were shown as a green box, orange box, or red box, respectively. Gray boxes are shown if the susceptibility to agents in that class is not assessed. The experiment is performed in triplicate. QZ18050: MDR A. baumannii, QZ18106: MDR K. pneumoniae, QZ18109: MDR E. coli, QZ19121- QZ19125: MDR P. aeruginosa.

**Table 4:** The MIC (μM) values of antimicrobial agents against *P. aeruginosa* PAO1 on different days.

Antimicrobial agents	Day 1	Day 150	Fold change in MIC
AS-hepc3_(41-71)_	8	12	1.5
AS-hepc3_(48-56)_	8	24	3
Ciprofloxacin	0.17	174.01	1024
Levofloxacin	0.31	241.31	768
Meropenem	0.29	292.56	1024
Imipenem	1.58	302.50	192
Gentamicin	0.33	15.93	48
Tobramycin	0.53	1.07	2
Ceftazidime	1.83	1873.50	1024
Cefepime	0.87	167.98	192
Aztreonam	9.19	2351.70	256
Piperacillin	7.73	7914.21*^a^*	1024
Polymyxin B	1.44	1.44	1
Colistin	0.71	0.71	1

^a^The last day is “Day 132”.

### AS-hepc3_(48-56)_ Inhibits MDR *P. aeruginosa* Lung Infection and Inflammation in Mouse Model

**Figure 6:**
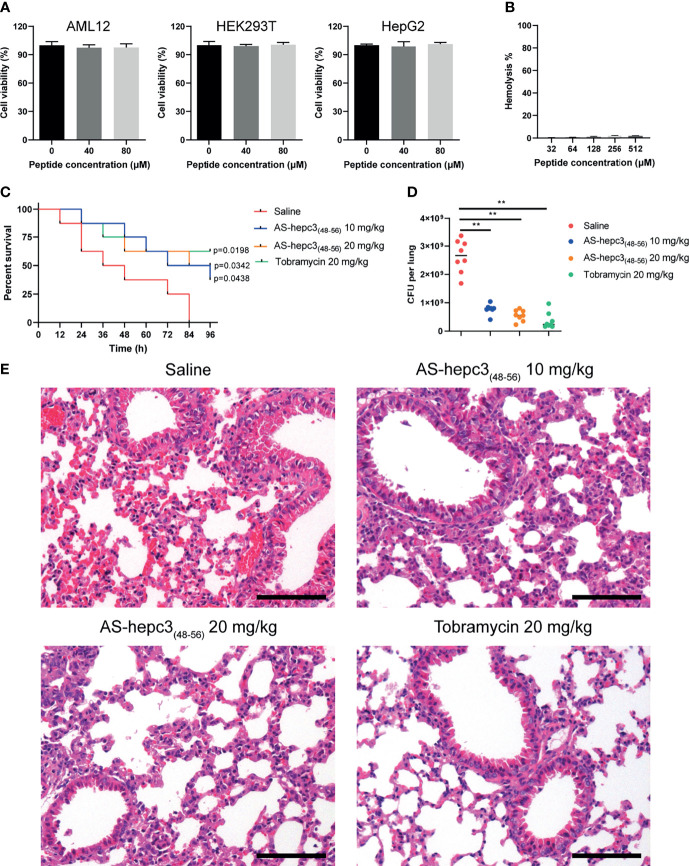
AS-hepc3_(48-56)_ inhibits MDR *P. aeruginosa* lung infection and inflammation *in vivo*. **(A)** The cytotoxicity of AS-hepc3_(48-56)_ towards human cell lines. **(B)** The hemolysis activity of AS-hepc3_(48-56)_ was assessed by incubating with mouse red blood cells. **(C)** Following intratracheal inoculation of mice (C57BL/6, male, n= 8) with MDR *P. aeruginosa* QZ19125 and subsequent intratracheal instillation of AS-hepc3_(48-56)_ or sterile saline to evaluate the survival rates by Kaplan–Meier survival curve. **(D)** The bacterial burden in lung homogenate was shown. **(E)** Representative histopathological images of processed lung section with hematoxylin and eosin (H&E) after treatment with sterile saline, AS-hepc3_(48-56)_, or tobramycin. Scale bars, 100 µm. Data are shown as means ± SD and statistical significance was determined by one-way ANOVA with Tukey’s multiple comparisons. **P < 0.01.

The *in vitro* safety was examined by the evaluation of the cytotoxicity and hemolytic activity of AS-hepc3_(48-56)_ towards human cell lines. Following the treatment of AS-hepc3_(48-56)_, the viability of AML12, HEK293T, and HepG2 cell lines was performed *via* MTS based cell viability assay. AS-hepc3_(48-56)_ exhibited no cytotoxicity, as the >95% of the mammal cells were viable following the treatment of AS-hepc3_(48-56)_ with 80 μM, a concentration of tenfold as high as the MIC against *P. aeruginosa* (**Figure 6A**). The hemolytic activity was evaluated by measuring the amount of hemoglobin released from healthy mouse red blood cells after treatment with AS-hepc3_(48-56)_ and the control. After incubation with 512 μM of AS-hepc3_(48-56)_, more than 98% of red blood cells kept integrity (**Figure 6B**). As we know, *in vivo* toxicity is critical for the assessment of therapeutic application. Therefore, we initially examined the toxicity of AS-hepc3_(48-56)_ to C57BL/6 mice by intratracheal (i.t.) instillation of up to 400 μg using mice weighing 20 g. All mice survived after 4 days of treatment. Next, we evaluated the *in vivo* efficacy of AS-hepc3_(48-56)_ against *P. aeruginosa* QZ19125 MDR clinical isolate (which is resistant to piperacillin, piperacillin/tazobactam, cefoperazone/sulbactam, ceftazidime, cefepime, imipenem, meropenem, aztreonam, ciprofloxacin, and levofloxacin), using a single intratracheal administration in a mouse pneumonia model. Mice were infected intratracheally with a minimum lethal dose of bacteria within 4 days. One hour after infection, AS-hepc3_(48-56)_ was administered intratracheally at a single dose of 20 mg/kg. Half of the treated mice were survived after 96 h, while all mice in the untreated group died (**Figure 6C**). AS-hepc3_(48-56)_ treatment significantly reduced the bacterial load in the lung homogenates (**Figure 6D**). Consistent with the bacteria load, histopathological examination of lung sections showed that the levels of inflammatory infiltrate and red blood cells in alveolar walls in mice treated with AS-hepc3_(48-56)_ were markedly reduced (**Figure 6E**).

## Discussion

Due to the increased resistance to most clinical antibiotics, *P. aeruginosa* infection has become a worldwide health problem. There is an urgent need to develop new therapeutic agents, and AMPs are thought to be potential ideal antimicrobial agents that could substitute the use of certain antibiotics. In the study, we reported two synthetic truncated peptides AS-hepc3_(41-71)_ and AS-hepc3_(48-56)_ with potent anti-*P. aeruginosa* activity, and in particular, they showed high activities against 5 strains of newly clinical isolated MDR *P. aeruginosa*. A long-term comparative study on the bacterial resistance treated with AS-hepc3_(41-71)_, AS-hepc3_(48-56),_ or 12 commonly used antibiotics indicated that the shortest peptide AS-hepc3_(48-56)_ would be a promising agent to be used for the treatment of *P. aeruginosa* infection clinically in future.

At the beginning of the study, four truncated peptides (AS-hepc3_(41-71)_, AS-hepc3_(52-71)_, AS-hepc3_(41-51)_, and AS-hepc3_(48-56)_) were evaluated on their antibacterial activity against *P. aeruginosa* PAO1. It was found that AS-hepc3_(41-71)_ exhibited potent activity against *P. aeruginosa* PAO1, while the other peptides AS-hepc3_(52-71)_ and AS-hepc3_(41-51)_ could not inhibit the growth of PAO1 even at the concentration of 128 μM. Considering more feasible applications in the future, a shorter peptide AS-hepc3_(48-56)_ was further explored. AS-hepc3_(48-56)_ is a cysteine- and arginine-rich peptide. The antibacterial activity of AS-hepc3_(48-56)_ was similar to that of AS-hepc3_(41-71)_. The cost of synthesizing AS-hepc3_(48-56)_ is much lower than AS-hepc3_(41-71)_, which is beneficial to future applications.

From the study, we noted that during 150 days of successive culture generations, *P. aeruginosa* quickly generated resistance to 9 antibiotics, however, the degree of resistance to each antibiotic was different. Among them, especially four antibiotics (including ceftazidime (MIC for the 150th day (μg/mL)/MIC for the first day (μg/mL), 1024/1)), meropenem (128/0.125), ciprofloxacin (64/0.0625), and piperacillin (4096/4)), their antibacterial activity decreased sharply, and the effective dose of the 150th day was 1000 times higher than that of the first day. A previous study shows that under long-term continuous exposure to antibiotics, *P. aeruginosa* could induce adaptive resistance due to mutations in the resistant genes (Botelho et al., 2019). As it is known, *P. aeruginosa* can perform multiple mechanisms to adapt to the stress of antibiotics, including reduced antibiotic uptake, modifications of antibiotic targets, overexpression of efflux pumps, overexpression or acquisition of antibiotic-inactivating enzymes, and formation of biofilm and persister cells (Wyres and Holt, 2018). In particular, *P. aeruginosa* is a Gram-negative bacterium, its outer membrane can act as a permeation barrier, preventing the entry of cytotoxic molecules including antibiotics and AMPs (Ebbensgaard et al., 2018; Vetterli et al., 2018). In contrast, *P. aeruginosa* did not generate any resistance to polymyxin B and colistin, and its resistance to the other six antibiotics was relatively low. The discrepancy in resistance generated by *P. aeruginosa* among 12 antibiotics might be related to the different resistant mechanisms to different classes of antibiotics. As reported, ciprofloxacin and levofloxacin belong to fluoroquinolones which interfere with DNA replication by interacting with DNA gyrase and topoisomerase IV. Mutations in genes encoding DNA gyrase and/or topoisomerase IV can decrease the binding affinity of relative proteins to fluoroquinolones, leading to resistance to fluoroquinolones in *P. aeruginosa* (Bruchmann et al., 2013). To enter the cell, β-lactams and fluoroquinolones penetrate the cell membrane through porin channels. The mutation of relative porin genes could decrease the penetration of both of these two kinds of antibiotics leading to antibiotic resistance. Gentamicin is an aminoglycoside antibiotic that inhibits protein synthesis by targeting the 30S ribosomal subunit. Ribosomal mutations in *P. aeruginosa* can induce a high level of resistance to aminoglycosides (Sanz-Garcia et al., 2018). Conversely, both polymyxin B and colistin maintained their anti-*P. aeruginosa* activity for 150 days without any change of antibacterial concentration. Both antibiotics belong to polymyxins that are usually interacted with the negatively charged phosphate residues of lipid A on the cell membrane and disrupt the outer membrane of Gram-negative bacteria (Ben Jeddou et al., 2020). The structural remodeling of lipid A is the primary cause of polymyxin resistance (Zhang et al., 2019). While the evolution of colistin resistance is complex and has multiple steps, which makes it hard for *P. aeruginosa* to develop resistance to colistin (Jochumsen et al., 2016). In addition, compared with the intracellular targeting antibiotics, membrane targeting antibiotics, such as colistin, killed the bacteria quickly (>99% of bacteria were killed in 1 h at 2 μg/mL) (Li et al., 2006), which might be the reason why *P. aeruginosa* is hard to develop resistance to membrane targeting antibiotics.

Correspondingly in the study, *P. aeruginosa* did not develop significant resistance after 150 days of continuous treatment at sub-inhibitory concentrations of AS-hepc3_(41-71)_ and AS-hepc3_(48-56)_. In the long-term efficacy experiment on *P. aeruginosa* PAO1, various antibiotics were used as comparators, especially colistin and polymyxin B. The MIC value of polymyxin B is 2 μg/mL (~1.44 μM), and the value of colistin is 2 μg/mL (~0.71 μM). After 150 days of continuous passage, colistin and polymyxin B did not develop resistance, and the MIC values were still 2 μg/mL (1.44 μM) and 2 μg/mL (0.71 μM), respectively. The MIC value of AS-hepc3_(41-71)_ and AS-hepc3_(48-56)_ on the first day is 8 μM. After 150 days of continuous passage, the MIC value of AS-hepc3_(41-71)_ increased from 8 μM to 12 μM, and AS-hepc3_(48-56)_ increased from 8 μM to 24 μM, as shown in **Table 4**. Both AS-hepc3_(41-71)_ and AS-hepc3_(48-56)_ exhibited similar anti- *P. aeruginosa* activity with LL-37 which is a widely studied peptide. LL-37 shows a broad spectrum against both Gram-negative pathogens, such as *A. baumanii*, *E. coli*, and *P. aeruginosa*, and Gram-positive pathogens, such as *S. aureus* and *S. epidermidis in vitro* (Feng et al., 2013; Xhindoli et al., 2016; de Breij et al., 2018). Like LL-37, AS-hepc3_(48-56)_ also showed broad-spectrum activity. The evaluation of LL-37 in clinical trials has been Phase IIb in venous leg ulcers (Mookherjee et al., 2020). The peptide AS-hepc3_(48-56)_ showed similar antibacterial activity and mechanism to LL-37 against *P. aeruginosa*, suggesting that AS-hepc3_(48-56)_ was a potential alternative to antibiotic in the treatment of topical infection

Polymyxins, such as colistin and polymyxin B, mainly target Gram-negative bacteria (Falagas and Kasiakou, 2005). The main mechanism of polymyxins is mainly to attack the cell membrane of Gram-negative bacteria. Due to its positive charge, polymyxins interact with the negatively charged phosphate groups of lipid A in the outer membrane of Gram-negative bacteria, disrupting membrane permeability, and ultimately leading to cell death (Poirel et al., 2017). In addition, some alternative mechanisms of action have been reported, such as inhibiting the activity of important respiratory enzymes and ribosome binding (Trimble et al., 2016). In the long-term of serial passage experiments, *P. aeruginosa* PAO1 did not develop significant resistance to our peptides, which suggested that they may have similar mechanisms of action. In a previous study, researchers found that the treatment of polymyxin B or colistin causes *P. aeruginosa* cell membranes damage and the release of cytoplasm contents (Falagas and Kasiakou, 2005).

Based on the imaging observation, both peptides were at first attached to the outer membrane of *P. aeruginosa* due to electrostatic interaction, afterwards the damage was observed on the bacterial cells. The damage on cells would increase the permeability of the outer membrane that could accelerate the uptake of antimicrobial agents into the cytoplasm as detected in our study. Previous studies have reported that AMPs could destroy bacterial membranes or interact with the intracellular targets to kill microorganisms (Brogden, 2005; Scocchi et al., 2016). For example, crotalicidin and magainin 2, have been shown to inhibit or kill various pathogens by inducing membrane permeabilization (Hasan et al., 2018; Pérez-Peinado et al., 2018). In addition, buforin II and Bac7 exert antimicrobial activity by inhibiting protein folding or enzymatic activity, or through intracellular effects (Bustillo et al., 2014; Mishra et al., 2017). Our present study showed that peptides possess different mechanism of action by disturbing membrane permeability and acting with the intracellular component compared with other AMPs, which might make it difficult for bacteria to develop resistance.

Our results seemed to the antibacterial mechanism of polymyxins, and these cationic AMPs interacted with the bacterial cell membrane and increased membrane permeability would lead to cell death. AMPs are difficult to induce bacterial resistance due to the rapid bactericidal effect and membrane-based mechanism of action (Zasloff, 2002). Accordingly, the clinical MDR *P. aeruginosa* is difficult to generate resistance to AS-hepc3_(48-56)_ that makes the 9- amino acid shorter peptide an ideal drug candidate for further clinical therapy.

*In vivo* evaluation of AMPs against clinical MDR bacteria is very important for future application. Many synthetic AMPs have been evaluated in preclinical studies and clinical trials (Mookherjee et al., 2020). A recent study shows that the topical application of SAAP-148, an LL37-derived peptide, effectively eradicates infections with MDR *A. baumannii* from wounded murine skin *in vivo* (de Breij et al., 2018). Similarly, administration of a synthetic peptide IK8L shows efficacy on burn wounds of mice caused by the MDR *P. aeruginosa* infection (Zhong et al., 2017). Otherwise, administration of a synthetic peptide ZY4 effectively has inhibited MDR *P. aeruginosa* lung infection and inflammation *in vivo* and suppressed dissemination of MDR *P. aeruginosa* and *A. baumannii* in a mouse septicemia infection model (Mwangi et al., 2019). It was interesting to note in the study that a dose of 20 mg/kg AS-hepc3_(48-56)_ showed a significant inhibition on the MDR *P. aeruginosa* infection about 5-fold which was similar to the efficacy of tobramycin at the same dose. The reduction of bacteria load represented a fact that the short peptide could significantly alleviate the inflammatory response caused by *P. aeruginosa*, thus indicating that AS-hepc3_(48-56)_ might be useful *in vivo* to inhibit the *P. aeruginosa* infection. In the clinic, MDR *P. aeruginosa*-related pneumonia can cause increased nosocomial mortality compared to non-MDR infection (Micek et al., 2015). The administration of AS-hepc3_(48-56)_ significantly increased the survival rate of mice with MDR *P. aeruginosa* lung infection by 50% than controls and these results demonstrated that AS-hepc3_(48-56)_ possessed therapeutic efficacy in MDR *P. aeruginosa* infection. AS-hepc3_(48-56)_ also has a potential efficacy on the infection of the other MDR pathogens in topical administration, such as MDR *A. baumannii*, MDR *K. pneumoniae*, and MDR *E*. *coli*. In our previous study, the synergistic effect of an AMP Sph_12-38_ (a truncated peptide from Sphistin) with antibiotics (azithromycin and rifampicin) showed a significant efficacy on a mouse skin infection model (Ma et al., 2017; Liu et al., 2020). AS-hepc3_(48-56)_ showed a similar mechanism of action to Sph_12-38_ which can cause the permeabilization of bacteria membrane (Ma et al., 2017). In a previous study, some combinations between AMPs and conventional antibiotics showed a synergistic effect against MDR *P.aeruginosa* (Ruden et al., 2019). Whether AS-hepc3_(48-56)_ may exhibit synergy with antibiotics against some MDR pathogens deserves further investigation.

In summary, the synthetic short peptide AS-hepc3_(48-56)_ derived from AS-hepc3_(41-71)_ retained the strong antimicrobial activity of AS-hepc3_(41-71)_ and showed a similar antimicrobial mechanism to AS-hepc3_(41-71)_. Compared with 12 selected clinical antibiotics, both AS-hepc3_(41-71)_ and AS-hepc3_(48-56)_ could maintain the effective inhibition at a stable dose on *P. aeruginosa* growth after 150 days of continuous treatment without inducing resistant mutations. Under certain experimental conditions, the antibacterial mechanism of AS-hepc3_(48-56)_ was demonstrated to change the permeability of the outer and inner membranes, destroy the membrane integrity, cause the leakage of cytoplasmic components, and ultimately result in the death of *P. aeruginosa*. Otherwise, AS-hepc3_(48-56)_ had potent antimicrobial activity against clinical isolates of MDR *P. aeruginosa*. and had a therapeutic effect on the MDR *P. aeruginosa* infection in mice. The present study provided a promising candidate for topical applications in MDR *P. aeruginosa* infection clinically.

## Data Availability Statement

The original contributions presented in the study are included in the article/supplementary material. Further inquiries can be directed to the corresponding author.

## Ethics Statement

The animal study was reviewed and approved by Laboratory Animal Management and Ethics Committee of Xiamen University (XMULAC20210029).

## Author Contributions

DZ: Data curation, Formal analysis, Investigation, Methodology, Writing - original draft. FC: Project administration, Supervision, Writing - review & editing. Y-CC and HP: Methodology. K-JW: Funding acquisition, Project administration, Supervision, Writing - review & editing. All authors contributed to the article and approved the submitted version.

## Funding

This study was supported by the Fujian Marine Economic Development Subsidy Fund Project (grant # FJHJF-L-2019-1) from the Fujian Provincial Department of Ocean and Fisheries, the Xiamen Ocean and Fishery Development Special Fund Project (grant # 20CZP011HJ06) from the Xiamen Municipal Bureau of Ocean Development, a grant (grant # 3502Z20203012) from the Xiamen Science and Technology Planning Project from Xiamen Municipal Bureau of Science and Technology, and the Fundamental Research Funds from Central Universities (grant # 20720190109).

## Conflict of Interest

The authors declare that the research was conducted in the absence of any commercial or financial relationships that could be construed as a potential conflict of interest.

## Publisher’s Note

All claims expressed in this article are solely those of the authors and do not necessarily represent those of their affiliated organizations, or those of the publisher, the editors and the reviewers. Any product that may be evaluated in this article, or claim that may be made by its manufacturer, is not guaranteed or endorsed by the publisher.
